# GBS and COVID-19: Untangling the Knots

**DOI:** 10.1017/cjn.2021.128

**Published:** 2022-01

**Authors:** Monica Alcantara, Hans D. Katzberg

**Affiliations:** University of Toronto, Neurology, 200 Elizabeth St. 5 EC-309, Toronto, Ontario M5G 2C4, Canada; Toronto General Hospital/UHN – Neurology, 200 Elizabeth St., Toronto, Ontario M5G 2C4, Canada

**Keywords:** GBS, COVID-19, SARS-COVID-19, Guillain–Barre

In this issue of the *Canadian Journal of Neurological Sciences*, Aladawi et al. describe through a systematic review, a series of patients with Guillain–Barre syndrome (GBS) who presented with evidence of recent COVID-19 infection in the article “Guillain Barre Syndrome as a Complication of COVID-19: A Systematic Review.”^1^ Following the Preferred Reporting Items for Systematic Reviews and Meta-Analyses (PRISMA) statement,^2^ the authors identified 1,450 records and included 81 studies comprising 109 patients in total. This interesting work included patients diagnosed according to Brighton Collaboration Criteria^3^ and excluded patients with preceding infections other than SARS-CoV2, alternative diagnosis for weakness and latency period between COVID-19 infection and GBS symptoms beyond 6 weeks.

COVID-19 infection was confirmed in 99 cases with either PCR testing or serology and 77/99 fulfilled a high level of diagnostic certainty for GBS diagnosis (Brighton Criteria 1 or 2), after excluding Miller–Fisher syndrome (MFS) and other variants. GBS cases included classical sensorimotor variants in most patients (64 cases), paraparetic GBS in 16, MFS in 9, and other variants (facial diplegia with paresthesia, pharyngeal–cervical–brachial GBS, and pure sensory GBS) in the remainder. Nerve conduction studies done showed 59/77 acute inflammatory demyelinating polyneuropathy (AIDP), 8/77 acute motor axonal neuropathy, and 10/77 acute motor and sensory axonal neuropathy.

These results parallel data from case series, reviews, cohort, and population-based studies including COVID-19/GBS patients and GBS cases prior to the pandemic, which showed similar age of onset, male predominance, and electrophysiologic profile.^4–8^ The authors reported an outstanding male predominance in COVID-19/GBS patients (male: female ratio of 2.5:1) Increased age and male gender are also known risk factors for severe COVID-19 outcomes, including hospitalizations and death, which could reflect the higher prevalence of GBS in hospitalized male patients.^9^ The most frequent electrophysiologic profile in both COVID-19-related GBS and GBS from other causes is AIDP, making up at least 60% of the cases in most series.^1,4,5^ In a well-designed cohort study, the predominant electrophysiological subtype was demyelinating in Europe/Americas (55%), while axonal forms predominate in Asian countries, reflecting disparities in clinical presentation.^10^

Much remains unknown about the strength of the association of GBS and COVID-19 pandemic as compared to prior epidemics/pandemics. For instance, during the Zika virus outbreaks in French Polynesia, a case–control study provided strong evidence of a causative link between the virus and GBS.^11^ In contrast, Keddie and colleagues did not find a similar strong causal link between COVID-19 infection and GBS during the first wave of the pandemic in a well-designed UK epidemiological and prospective cohort study using intravenous immunoglobin administration data and available clinical records.^6^ Further adding to the uncertainty is the lack of homology found in COVID-19 proteins and human nerve axonal or myelin proteins and glycoproteins.^6^ Although the current study made attempts to exclude patients with alternate infectious triggers and those with prolonged latency between COVID-19 infection and GBS, further insights on the strength of the association, causal link, or true incidence are not possible due to the limited case descriptions and lack of epidemiological perspective in the current report. In another study, some patients had GBS symptoms while COVID-19 symptoms were still ongoing, raising the possibility of a direct infectious process in some cases; however, the high rates of albumin-cytologic dissociation in most reports including this one suggests a postinfectious inflammatory process.^12^ It should also be noted that reports have indicated that GBS rates overall have been lower than usual since the pandemic began, hypothesized to be related to isolation, distancing and hygiene public health measures instituted in many regions.^6^

In the current study, the authors pointed out that COVID-19-related GBS could be associated with a severe disease course with frequent intensive care unit/ventilation requirements. The proportion of GBS/COVID-19 patients who required mechanical ventilation in their study (30%) was similar to prior studies.^5,6,8^ One should again be cautious when interpreting those figures, as in a study comparing data with pre-pandemic GBS historical cohort, the difference in proportions of patients requiring mechanical ventilation did not reach statistical significance.^6^ Furthermore, as COVID-19 hospitalized patients with severe pulmonary disease are more likely to be ventilated, confounders and bias in ascertaining risk factors could overestimate causal effects.

Most of the cases here described in this report pertained to European Union countries (66/99), which could represent a degree of reporting bias, as there might be a higher likelihood of reporting severe cases requiring hospitalization/investigations in specialized centers. In addition, cases are more likely to be underreported in underdeveloped nations due to specialized diagnostic requirements (electrophysiologic studies and cerebrospinal fluid analysis) which may be lacking due to limited resources.^3,13^

Further developments are expected in the following months on reports of GBS in the setting of new and recurrent COVID-19 as well as other types of infections which may increase as isolation restrictions are lifted. In addition, surveillance for immune reactions including GBS will be ongoing as COVID-19 vaccination efforts are rolling out in Canada. There are currently two mRNA vaccines – Pfizer-BioNTech (BNT162b2) and Moderna (mRNA-1273), one adenovirus-vectored COVID-19 (Covishield/Astra-Zeneca ChAdOx1 vaccine) and a recombinant adenovirus serotype 26 (Janssen/Johnson & Johnson) vaccine approved by Health Canada. In the Janssen/Johnson & Johnson trial, two patients (one in placebo and one in the intervention arm) developed GBS around 10 days after the inoculation.^14^ In Canada, as of May 14, 2021, there have been 13 cases of GBS reported among 17,734,322 vaccine doses; however, these cases will require detailed case descriptions and epidemiological analysis not yet available before any causal links are confirmed.^15^ At the time of this writing, Canada and many countries around the world are still in the midst of the fight against COVID-19 and vaccination efforts have shown the best hope for ending the global pandemic. Going forward, this article and other similar reports evaluating the relationships between GBS and COVID-19 infection, as well as between GBS and COVID-19 vaccination, will continue to enhance our understanding of these important but likely rare occurrences.
